# Effects of nicorandil on PI3K/Akt signaling pathway and its anti-apoptotic mechanisms in coronary microembolization in rats

**DOI:** 10.18632/oncotarget.19966

**Published:** 2017-08-05

**Authors:** Qiang Su, Lang Li, Jinmin Zhao, Yuhan Sun, Huafeng Yang

**Affiliations:** ^1^ Department of Cardiology, The First Affiliated Hospital of Guangxi Medical University, Nanning, China; ^2^ Department of Trauma Orthopedic and Hand Surgery, The First Affiliated Hospital of Guangxi Medical University, Nanning, China; ^3^ Guangxi Key Laboratory of Regenerative Medicine, Guangxi Medical University, Guangxi, China

**Keywords:** nicorandil, coronary microembolization, myocardial injury, apoptosis, PI3K/Akt

## Abstract

Coronary microembolization (CME) is a common complication of percutaneous coronary intervention (PCI) for acute coronary syndrome. It leads to myocardial apoptosis and cardiac dysfunction. Nicorandil pretreatment can prevent PCI-related myocardial injury and reduce the incidence of no- or slow-reflow phenomena. This cardioprotective effect is probably attributable to the suppression of CME-induced cardiomyocyte apoptosis, but the specific mechanisms have not been clarified. We aimed to investigate the protective effects of nicorandil pretreatment on CME-induced myocardial injury and clarify the underlying mechanisms. *In vivo* studies, we used echocardiography, cardiac-enzymes measurement, hematoxylin–basic fuchsin–picric acid staining, TUNEL assay, and western blot, and found that CME significantly increased apoptotic cardiomyocytes in the infarct and peri-infarct areas in rats. The PI3K/Akt signaling pathway was involved in cardiomyocyte apoptosis. Nicorandil pretreatment given 7 days before CME effectively reduced cardiomyocyte apoptosis and myocardial injuries in rats, mainly through the activation of PI3K/Akt signaling. *In vitro* studies further showed that nicorandil reduced hypoxia-induced cardiomyocyte apoptosis and improved cardiomyocyte-survival rate. The PI3K-specific inhibitor LY294002 reduced these cardioprotective effects, indicating that they were attributable to the activation of the PI3K/Akt signaling pathway. In conclusion, nicorandil has significant cardioprotective effects in CME mainly through the activation of the PI3K/Akt signaling pathway and reduction of CME-induced cardiomyocyte apoptosis. Our findings may provide important support for the pre-PCI use of nicorandil to reduce post-PCI myocardial injuries.

## INTRODUCTION

Coronary microembolization (CME) is an intractable complication of percutaneous coronary intervention (PCI) for acute coronary syndrome. It occurs when atherosclerotic plaques detach and obstruct the distal microvessels, resulting in no- or slow-reflow phenomena. CME is an independent and strong predictor of long-term adverse outcomes and major adverse cardiovascular events [1, 2]. Many animal studies have shown that microembolic areas and necrotic/apoptotic cardiomyocytes appear during the acute phase of CME [3, 4]. Cardiomyocyte apoptosis plays a critical role in CME-induced cardiac systolic dysfunction, and the suppression of cardiomyocyte apoptosis reduces CME-induced myocardial injuries [5, 6]. The downregulation of *PTEN* gene expression significantly alleviates cardiomyocyte apoptosis and improves cardiac contractility mainly through the activation of the PI3K/Akt signaling pathway. Moreover, drugs such as atorvastatin, which upregulate the PI3K/Akt signaling pathway, can effectively reduce CME-induced cardiomyocyte apoptosis and the subsequent myocardial injuries.

Nicorandil, a nicotinamide nitrate, is a vasodilator and potassium channel opener. It not only dilates blood vessels by activating intracellular guanylate cyclase but also directly or indirectly activates the nitric oxide/protein kinase G signaling pathway and increases K^+^ outflow to cause hyperpolarization, resulting in reduced intracellular calcium movement and coronary arterial dilatation [7, 8]. Nicorandil also exerts cardioprotective effects by upregulating the PI3K/Akt signaling pathway and reducing endoplasmic reticulum stress-mediated cardiomyocyte apoptosis [9]. Recently, nicorandil was used to prevent post-PCI myocardial injuries. These beneficial effects of nicorandil are probably the result of improvements in endothelial function and anti-apoptotic effects [10-12]. Although the underlying mechanisms remain to be fully elucidated, it is hypothesized that the cardioprotective effects of nicorandil are associated with the improvement of CME-induced cardiac systolic dysfunction. This study aimed to determine the effects of nicorandil pretreatment on hypoxia-induced cardiomyocyte apoptosis and the PI3K/Akt signaling pathway in rats and *in vitro*, in order to uncover the mechanisms underlying the cardioprotective effects of nicorandil in CME. Our study may provide evidence supporting the use of nicorandil for the prevention and management of CME.

## RESULTS

### Changes in cardiac function

The cardiac function of rats was evaluated by measuring LVEF, FS, CO, and LVEDd at 6 h after CME (Table 1). Compared with the sham group, the CME group had significantly impaired cardiac contractility and left ventricular enlargement as indicated by decreased LVEF, FS, and CO and increased LVEDd (all *P* < 0.05). Compared with the CME group, the Nic group had better cardiac function as indicated by higher LVEF, FS, and CO and lower LVEDd (all *P* < 0.05), the Nic+LY group had similar cardiac function (*P* > 0.05), and the LY group had poor cardiac function (*P* < 0.05).

**Table 1:** Changes in cardiac function ($\bar{x}\pm s$)

Group	*n*	LVEF (%)	LVFS (%)	CO (L/min)	LVEDd (mm)
Sham	10	79.56 ± 3.37	42.06 ± 4.48	0.191 ± 0.024	5.65 ± 0.32
CME	10	62.81 ± 2.74*	21.37 ± 2.69*	0.101 ± 0.007*	7.81 ± 0.56*
Nic	10	75.53 ± 4.02^#^	39.02 ± 4.67^#^	0.169 ± 0.018^#^	5.94 ± 0.47^#^
Nic+LY	10	64.13 ± 2.66*	25.27 ± 2.83*	0.115 ± 0.011*	7.49 ± 0.61*
LY	10	54.86 ± 2.38*^#^	18.48 ± 3.04*^#^	0.094 ± 0.015*^#^	8.07 ± 0.72*^#^

LVEF, left ventricle ejection fraction; CO, cardiac output; LVEDd, left ventricular end-diastolic diameter; LVFS, left ventricle fractional shortening; CME, coronary microembolization; Nic, nicorandil; LY, LY294002.

^*^P < 0.05 compared with the sham group; ^#^*P* < 0.05 compared with the CME group.

### Serum cTnI level

**Figure 1:**
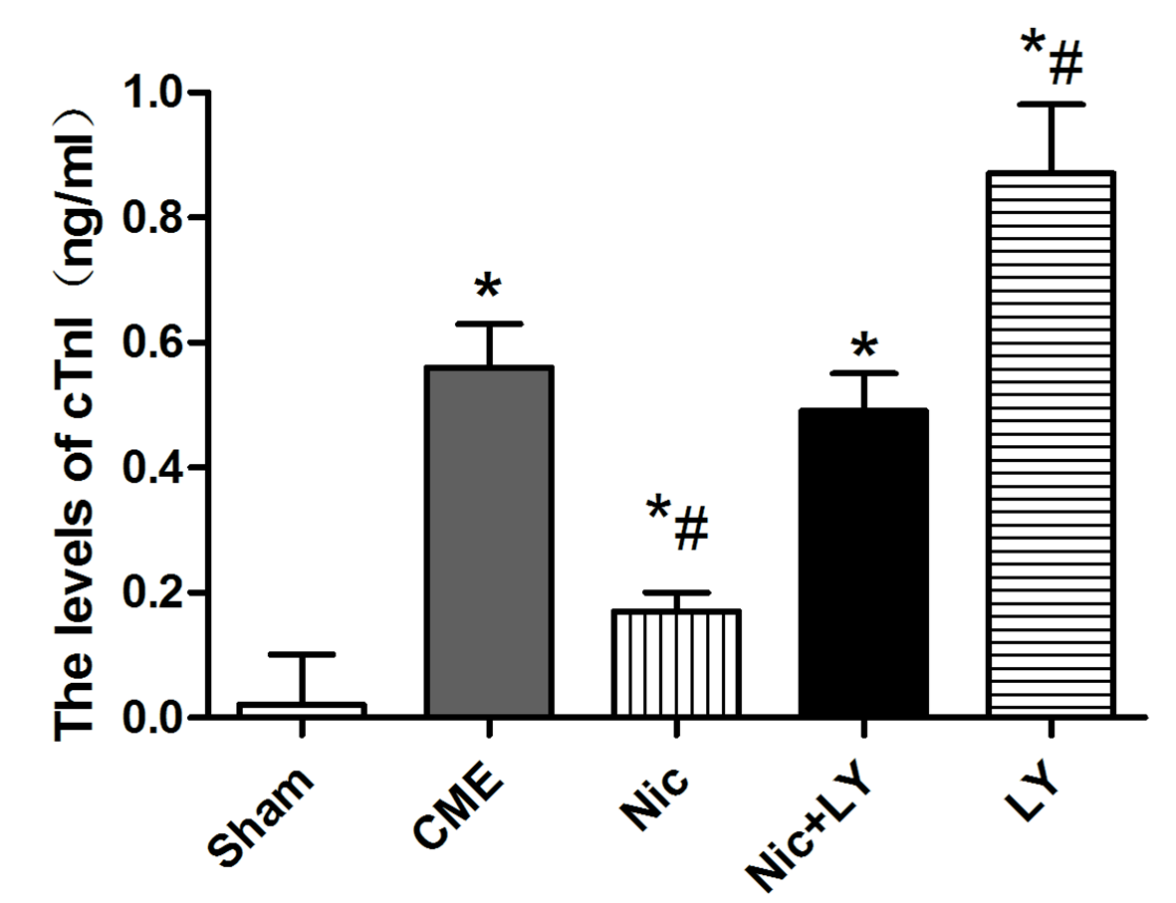
Serum cTnI levels in each group CME, coronary microembolization; Nic, nicorandil; LY, LY294002 ^*^P < 0.05 compared with the sham group; ^#^*P* < 0.05 compared with the CME group.

Compared with the sham group, the CME group had a significantly high serum cTnI level (0.56 ± 0.07 ng/mL *vs*. 0.021 ± 0.008 ng/mL, *P* < 0.05). Compared with the CME group, the Nic group had a significantly low serum cTnI level (0.17 ± 0.03 ng/ml, *P* < 0.05), the Nic+LY group had a similar serum cTnI level (0.49 ± 0.06 ng/mL, *P* > 0.05), and the LY group had a significantly high serum cTnI level (0.87 ± 0.11 ng/mL, *P* < 0.05; Figure 1).

### Pathological observation of CME

**Figure 2:**
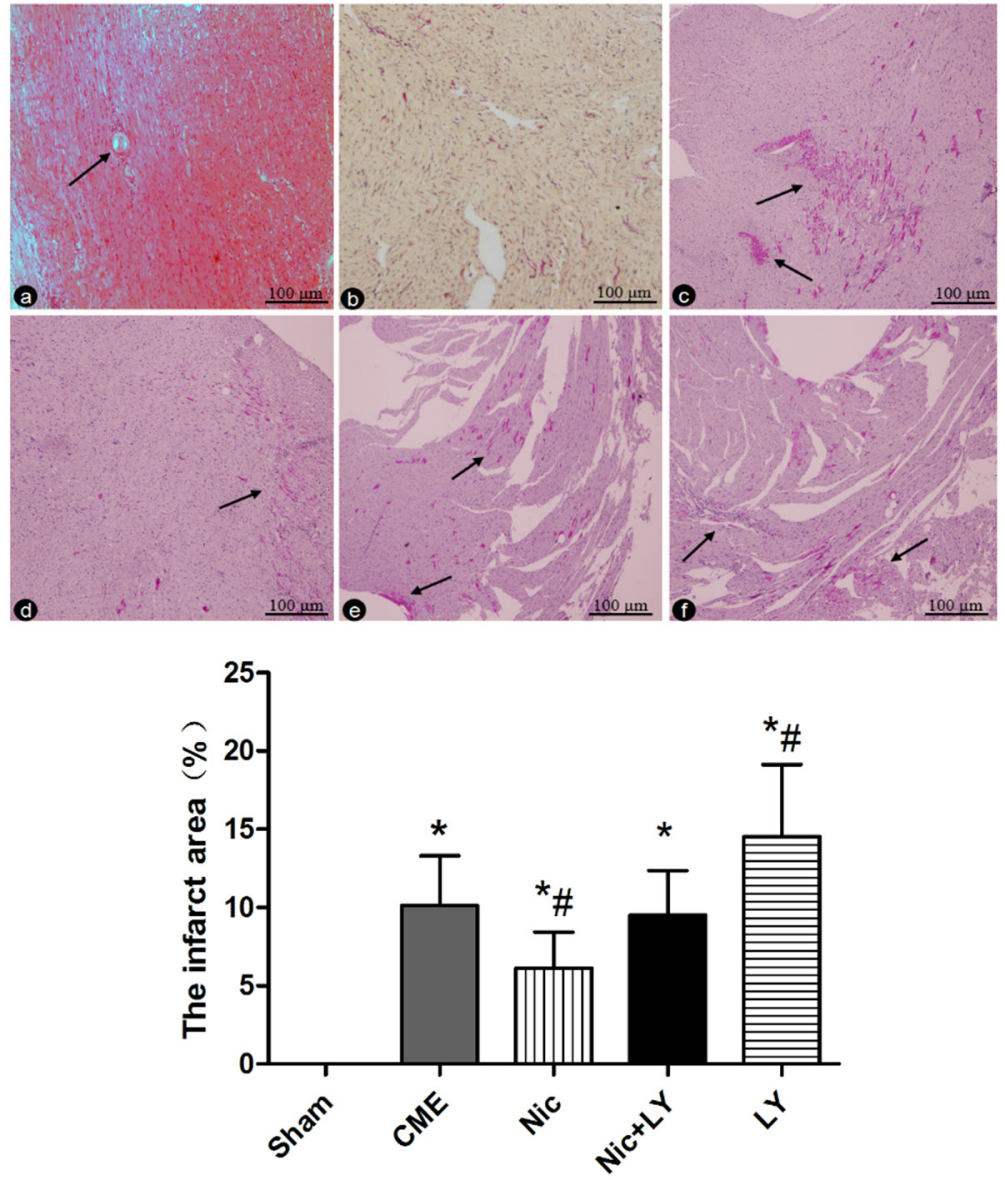
HE staining and HBFP staining **a.** HE staining of myocardial microinfarct areas in the CME group. The arrows indicate microspheres (×400 magnification). **b.-f.** HBFP staining in the sham group, CME group, Nic group, Nic+LY group, and LY group. Ischemic myocardium appears red. The arrows indicate microinfarct areas (×200 magnification). ^*^P < 0.05 compared with the sham group; ^#^*P* < 0.05 compared with the CME group.

HE staining and HBFP staining (Figure 2) showed sporadic subendocardial ischemic areas and no infarct areas in the sham group. The CME, Nic, Nic+LY, and LY groups had microinfarct areas, mostly in the shape of a wedge. The infarct areas were focally distributed in the subendocardium and left ventricle. HE staining showed that cardiomyocytes in the microinfarct areas had no nuclei or disrupted nuclei and red cytoplasm. Peri-infarct areas exhibited swollen and degenerated cardiomyocytes, inflammatory cell infiltration, and red blood cell exudation. Microspheres were observed in the microvessels. The infarct areas in the CME, Nic, Nic+LY, and LY groups were 10.14% ± 3.17%, 6.14% ± 2.29%, 9.51% ± 2.86%, and 14.53% ± 4.61%, respectively. Compared with the sham group, the CME group had a significantly larger infarct area (*P* < 0.05). Compared with the CME group, the Nic group had a significantly small infarct area (*P* < 0.05), the Nic+LY group had a similar infarct area (*P* > 0.05), and the LY group had a significantly large infarct area (*P* < 0.05).

### Effects of nicorandil on post-CME cardiomyocyte apoptosis in rats

**Figure 3:**
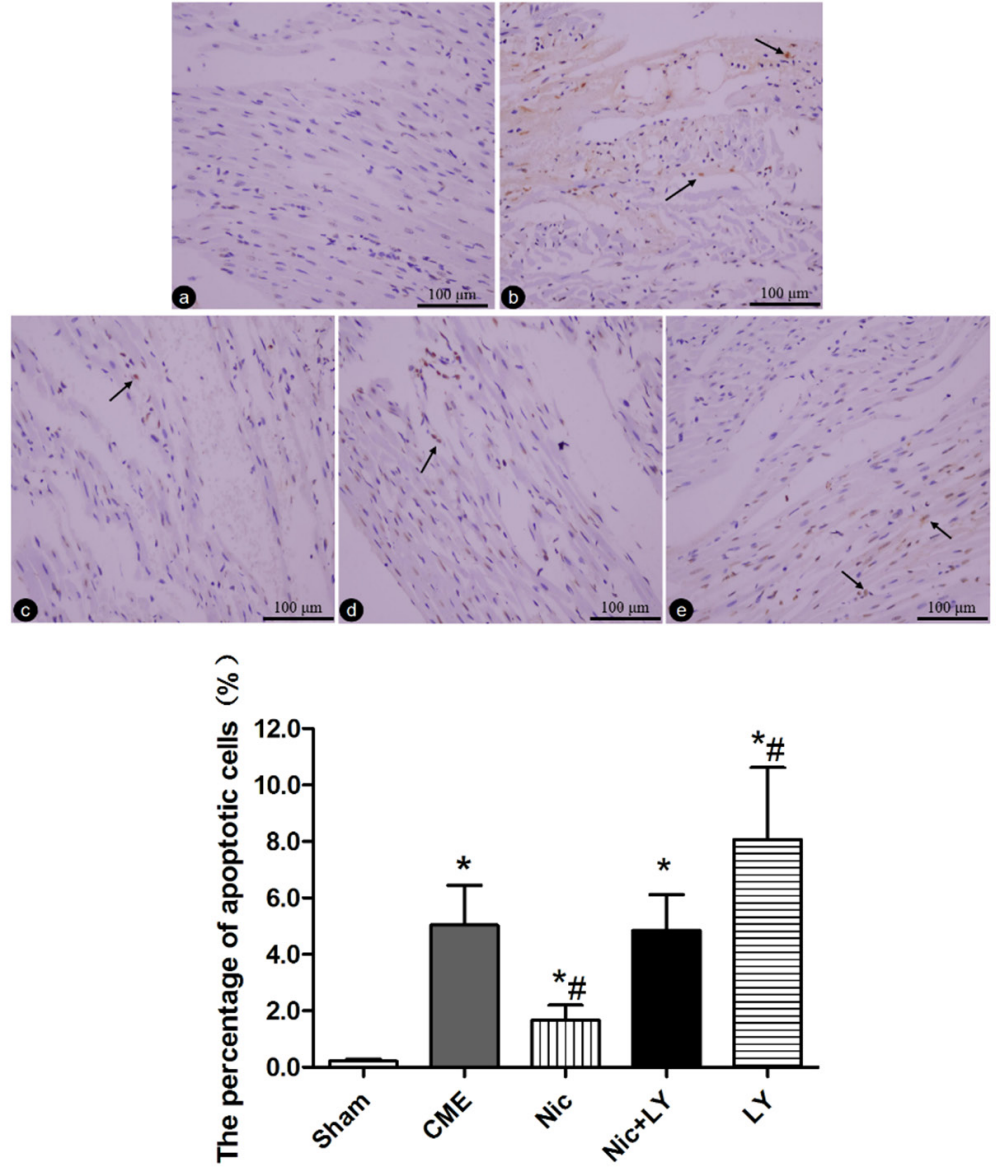
TUNEL staining of cardiomyocyte apoptosis **a.-e**. Sham group, CME group, Nic group, Nic+LY group, and LY group. The nuclei of apoptotic and normal cardiomyocytes appear yellow and light blue, respectively. The arrows indicate the nuclei of apoptotic cardiomyocytes (×400 magnification). * *P* < 0.05 compared with the sham group; ^#^*P* < 0.05 compared with the CME group.

*In situ* detection of cardiomyocyte apoptosis (Figure 3) showed that TUNEL-positive areas appeared yellow and light blue in the nuclei of infarcted and normal cardiomyocytes, respectively. Post-CME cardiomyocyte apoptosis was predominantly located in the myocardial microinfarct areas and peri-infarct areas. The cardiomyocyte-apoptosis rates in the sham, CME, Nic, Nic+LY, and LY group were 0.22% ± 0.07%, 5.04% ± 1.41%, 1.67% ± 0.53%, 4.84% ± 1.27%, and 8.06% ± 2.55%, respectively. Compared with the sham group, the CME group had a significantly high cardiomyocyte-apoptosis rate (*P* < 0.05). Compared with the CME group, the Nic group had a significantly low cardiomyocyte-apoptosis rate (*P* < 0.05), the Nic+LY group had a similar cardiomyocyte-apoptosis rate (*P* > 0.05), and the LY group had a significantly high cardiomyocyte-apoptosis rate (*P* < 0.05).

### Nicorandil reduces post-CME cardiomyocyte apoptosis through regulating the PI3K/Akt signaling pathway

**Figure 4:**
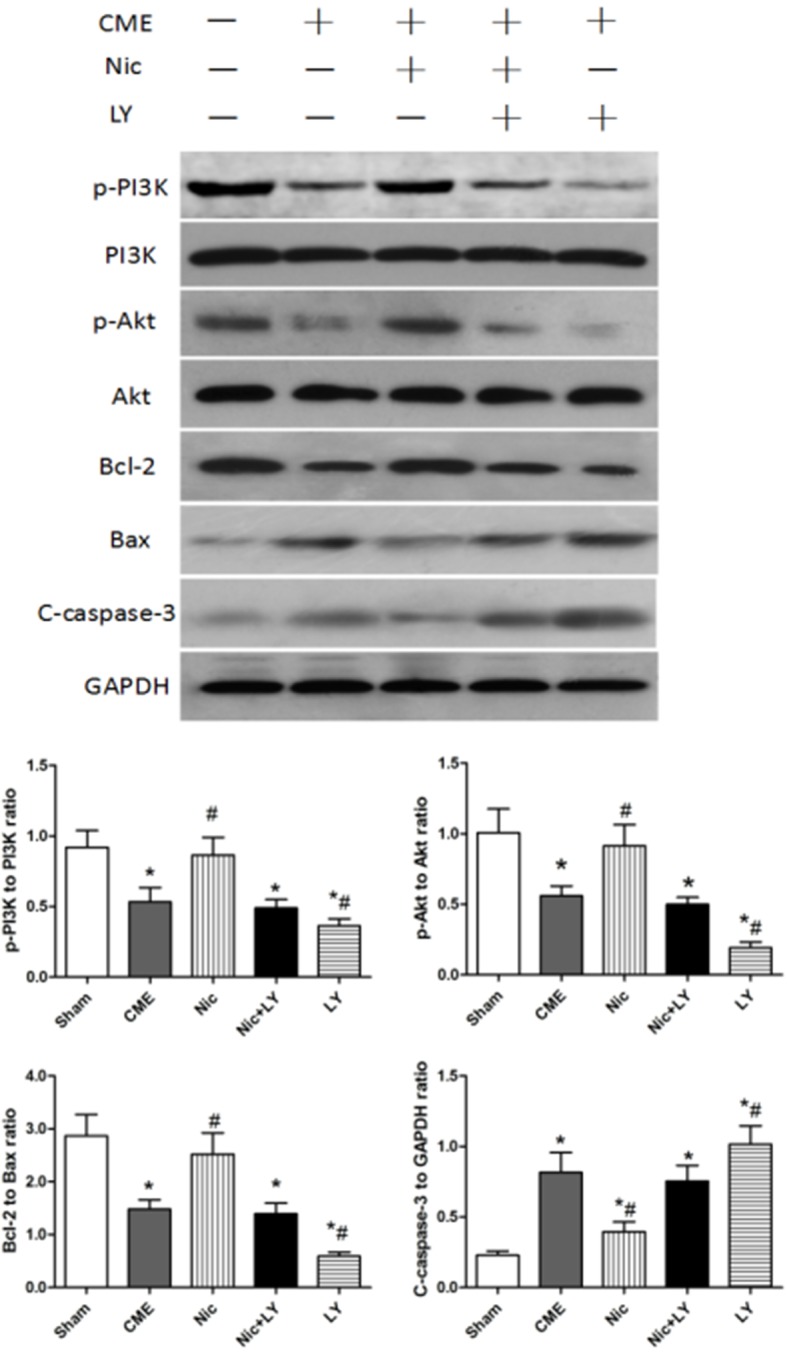
Effects of nicorandil on the PI3K/Akt signaling pathway and myocardial apoptosis in rats CME, coronary microembolization; Nic, nicorandil; LY, LY294002. ^*^P < 0.05 compared with the sham group; ^#^*P* < 0.05 compared with the CME group.

Western blot analysis (Figure 4) showed that compared with the sham group, the CME group had significantly low expressions of p-PI3K and p-Akt, which are the key proteins of the PI3K/Akt signaling pathway. In addition, the Bcl-2/Bax ratio was significantly downregulated and cleaved caspase-3 expression was upregulated in the CME group relative to the sham group (both *P* < 0.05). Compared with the CME group, the Nic group had significantly high expressions of p-PI3K and p-Akt, and a high Bcl-2/Bax ratio, but low expression of cleaved caspase-3 protein (all *P* < 0.05). The CME and Nic+LY groups did not significantly differ in terms of p-PI3K expression, p-Akt expression, Bcl-2/Bax ratio, and cleaved caspase-3 expression (all *P* > 0.05). Compared with the CME group, the LY group had significantly low p-PI3K and p-Akt expressions, a low Bcl-2/Bax ratio, and high cleaved caspase-3 expression (all *P* < 0.05). The above results indicated that the PI3K/Akt signaling pathway was involved in the post-CME myocardial apoptosis, and that nicorandil could activate this signaling pathway to reduce post-CME cardiomyocyte apoptosis.

### Effects of nicorandil on the survival rate of hypoxic cardiomyocytes

**Figure 5:**
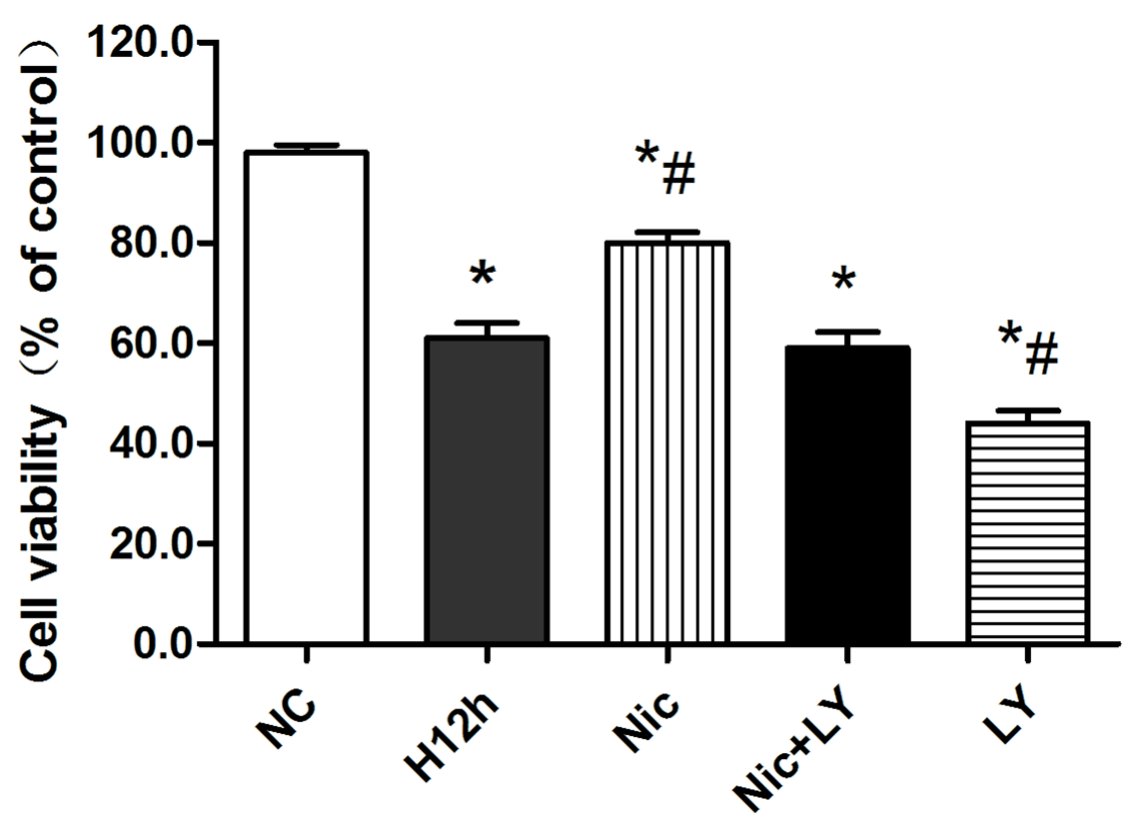
Effects of nicorandil on the survival rate of hypoxic cardiomyocytes NC, normoxic control; H12h, 12-h hypoxia; Nic, nicorandil; LY, LY294002 ^*^P < 0.05 compared with the sham group; ^#^*P* < 0.05 compared with the CME group.

As shown in Figure 5, the cardiomyocyte-survival rate was significantly reduced after 12 h of hypoxia (*P* < 0.05). This rate was significantly elevated by nicorandil pretreatment (*P* < 0.05). However, pretreatment with the combination of nicorandil and the PI3K-specific inhibitor LY294002 did not improve the survival rate (*P* > 0.05), indicating that the protective effects of nicorandil on hypoxic cardiomyocytes were blocked. The addition of LY294002 alone to the hypoxic cardiomyocytes resulted in a significant decrease in the cardiomyocyte-survival rate (*P* < 0.05).

### Nicorandil reduced hypoxia-induced cardiomyocyte apoptosis through regulating the PI3K/Akt signaling pathway

**Figure 6:**
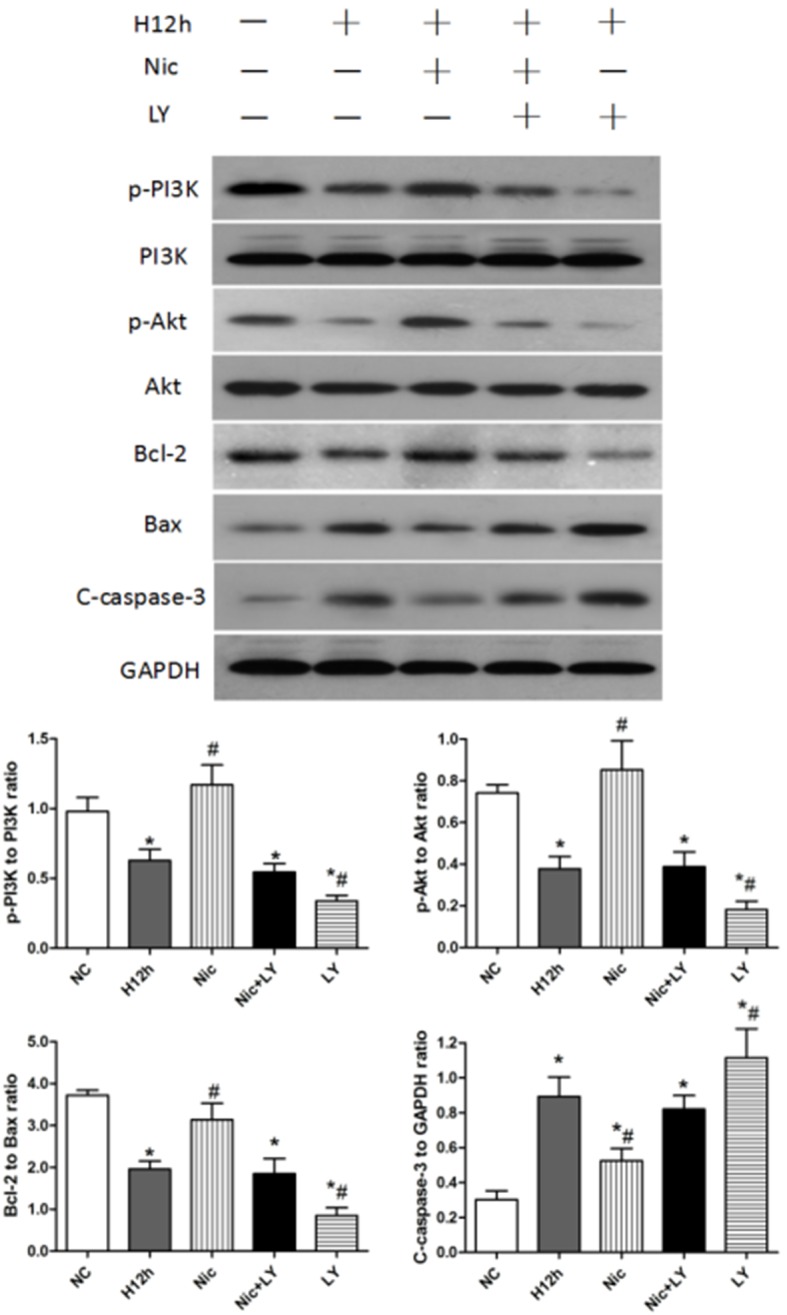
Effects of nicorandil on the PI3K/Akt signaling pathway and cardiomyocyte apoptosis in neonatal rats NC, normoxic control; H12h, 12-h hypoxia; Nic, nicorandil; LY, LY294002 ^*^P < 0.05 compared with the sham group; ^#^*P* < 0.05 compared with the CME group.

As shown in Figure 6, p-PI3K expression, p-Akt expression, and Bcl-2/Bax ratio were decreased after 12 h of hypoxia, but cleaved caspase-3 expression was significantly upregulated (all *P* < 0.05). When hypoxic cardiomyocytes were pretreated with nicorandil, p-PI3K expression, p-Akt expression, and Bcl-2/Bax ratio were significantly upregulated, and cleaved caspase-3 expression was downregulated (all *P* < 0.05). However, pretreatment with both nicorandil and LY294002 did not significantly alter p-PI3K, p-Akt, and cleaved caspase-3 expression or the Bcl-2/Bax ratio (all *P* > 0.05). Pretreatment with LY294002 alone exacerbated cardiomyocyte apoptosis, as indicated by significant downregulation of p-PI3K and p-Akt expression, and the Bcl-2/Bax ratio and significant upregulation of cleaved caspase-3 expression (all *P* < 0.05). These results showed that the PI3K/Akt signaling pathway is involved in hypoxia-induced cardiomyocyte apoptosis, and that nicorandil pretreatment can alleviate this apoptosis through regulating the PI3K/Akt signaling pathway.

## DISCUSSION

In this study, we found that cardiomyocyte apoptosis was increased in CME rats, and that the PI3K/Akt signaling pathway was involved in CME-induced cardiomyocyte apoptosis. Nicorandil pretreatment initiated 7 days before CME effectively reduced post-CME cardiomyocyte apoptosis and myocardial injury, mainly through the activation of the PI3K/Akt signaling pathway. Furthermore, *in vitro* studies proved that nicorandil could reduce hypoxia-induced cardiomyocyte apoptosis and increase the cardiomyocyte-survival rate, while the PI3K-specific inhibitor LY294002 could block the cardioprotective effects of nicorandil, indicating that these effects are mostly attributable to the activation of the PI3K/Akt signaling pathway in the hypoxic cardiomyocytes.

CME is commonly regarded as a spontaneous event in ischemic heart diseases or an iatrogenic complication during interventional treatments [13]. Unlike the proximal coronary obstruction in the epicardium, CME causes left ventricular systolic dysfunction that is not proportional to the size of the perfusion defect. Dörge et al. [14] injected microspheres into the coronary arteries of dogs and found that although coronary blood flow was transiently reduced, cardiac contractility was progressively impaired. In CME pigs and dogs, microinfarct areas account for less than 5% of the total myocardium [15]. The number of necrotic cardiomyocytes is so low that necrosis may not satisfactorily explain the progressive cardiac systolic dysfunction. Instead, the inflammatory response and cardiomyocyte apoptosis occurring in the peri-infarct areas play central roles in post-CME myocardial injury and progressive cardiac dysfunction [16, 17]. Our CME rat models showed elevated serum markers, exacerbated cardiac dysfunction, microinfarct areas, and apoptotic cardiomyocytes in microinfarct and peri-infarct areas, which are consistent with the pathophysiological changes of CME.

In studies of CME, significant cardiomyocyte apoptosis has been observed in infarct zones along with marked elevations of the apoptosis-related caspase-3 protein [6]. Multiple signaling pathways, including the PI3K/Akt signaling pathway, participate in the regulation of post-CME myocardial apoptosis, and their activity closely correlates with the progressive exacerbation of cardiac dysfunction [18, 19]. PI3K/Akt is a key intracellular signaling pathway involved in important physiological functions in cell survival, apoptosis, proliferation, etc. The activation of this pathway significantly reduces cardiomyocyte apoptosis and myocardial injury, while its inactivation leads to opposite effects [20-22]. In our study, we found that myocardial-apoptosis rates were significantly increased after CME. The PI3K/Akt signaling pathway participated in the regulation of cardiomyocyte apoptosis, and use of the PI3K-specific inhibitor LY294002 significantly suppressed the PI3K/Akt signaling pathway, reduced the Bcl-2/Bax ratio, upregulated cleaved caspase-3 expression, increased cardiomyocyte apoptosis, and exacerbated cardiac dysfunction. Nicorandil pretreatment markedly inhibited CME-induced cardiomyocyte apoptosis, alleviated myocardial injury, and improved cardiac function.

As the first ATP-sensitive potassium-channel opener approved for clinical use, nicorandil has nitrate-like activity and potassium channel-opening activity. It has been used to treat all types of angina pectoris, including exertional angina and vasospastic angina, to markedly reduce cardiovascular events [23, 24]. Recently, nicorandil has been used to prevent post-PCI myocardial injury and reduce no-reflow and slow-reflow phenomena. These cardioprotective effects are likely linked with the reduction of cardiomyocyte apoptosis [25-27]. Wu et al. found that nicorandil pretreatment significantly improved cardiac contractility and decreased infarct size in ischemia-reperfusion rat models. The underlying mechanisms were associated with upregulation of the PI3K/Akt signaling pathway and the subsequent reduction of endoplasmic reticulum stress-mediated cardiomyocyte apoptosis [9]. Zhang et al. [28] demonstrated that nicorandil reduced hypoxia-induced apoptosis in mesenchymal stem cells in rats, predominantly through activating the PI3K/Akt signaling pathway. As a common complication of PCI, CME induces myocardial apoptosis and subsequent myocardial injury. Therefore, the protective effects of nicorandil are likely the result of suppression of CME-induced myocardial apoptosis. In this study, we found that nicorandil could effectively reduce post-CME myocardial injury, reduce microinfarct areas, and improve cardiac function by suppressing cardiomyocyte apoptosis. Detection of the key proteins of the PI3K/Akt signaling pathway showed that nicorandil pretreatment could upregulate p-PI3K and p-Akt expressions. Following the use of the PI3K-specific inhibitor LY294002, the PI3K/Akt signaling pathway was suppressed, the cardioprotective effects of nicorandil disappeared, myocardial apoptosis significantly increased, and cardiac dysfunction was exacerbated. These findings indicate a major role of the PI3K/Akt signaling pathway activation in the cardioprotective effects of nicorandil against CME-induced myocardial injury.

To further confirm that the protective effects of nicorandil against CME-induced myocardial injury were associated with the regulation of the PI3K/Akt signaling pathway, we performed *in vitro* studies of hypoxic cardiomyocytes, which mimic CME. We found that nicorandil effectively improved the survival rate of hypoxic cardiomyocytes by reducing cardiomyocyte apoptosis. Furthermore, this protective effect was blocked by the PI3K-specific inhibitor LY294002, indicating that the nicorandil-induced reduction in hypoxia-induced cardiomyocyte apoptosis was very likely attributable to the upregulation of the PI3K/Akt signaling pathway.

In conclusion, nicorandil plays a significant cardioprotective role in CME-induced myocardial injury, mainly through activating the PI3K/Akt signaling pathway and subsequently reducing cardiomyocyte apoptosis. These mechanisms may be crucial to the pre-PCI use of nicorandil to reduce post-PCI myocardial injury.

## MATERIALS AND METHODS

### Animal preparation

All experimental protocols and procedures were approved by the Institutional Animal Care and Use Committee at Guangxi Medical University and were performed in compliance with the National Institutes of Health Guidelines on the Use of Laboratory Animals. Fifty male Sprague-Dawley rats (200-250 g) were obtained from the Medical Experimental Animal Center of Guangxi Medical University. All animals were housed under a 12-h light/dark cycle in a room maintained at a temperature of 23°C ± 2°C and humidity of 50%-60%, and were allowed free access to standard rat food and tap water.

### CME modeling and grouping

According to previous protocols [29], rats were anesthetized with an intraperitoneal injection of 30-40 mg/kg pentobarbital hydrochloride and intubated using tracheotomy. Small-animal ventilators were used to assist respiration. Thoracotomy was performed between the third and fourth ribs on the left side. The ascending aorta was isolated and clamped for 10 s when three thousand 42-μm microspheres (BioSphere Medical Inc., Rockland, USA) suspended in 0.1 mL sodium dodecyl sulfate were injected *via* the cardiac apex. The chest was closed, and the tracheal cannulas were removed. The rats were then intraperitoneally injected with 800,000 IU penicillin. The rats in the sham group were subjected to the same procedure, except that they were administered 0.1 mL normal saline instead of the microspheres. Fifty SD rats were randomized using a computer-generated table to the sham group, CME group, CME + nicorandil group (Nic), CME + nicorandil + LY294002 group (Nic+LY), and CME + LY294002 group (LY). Each group contained 10 rats. The rats in the Nic group were intragastrically administered nicorandil (Chugai Pharmaceutical Co., Japan) 15 mg/kg/d for 7 d before CME. The LY-group rats were intraperitoneally injected with LY294002 (Sigma-Aldrich, St. Louis, USA) 10 mg/kg at 30 min before CME. The rats in the Nic+LY group were intragastrically administered nicorandil 15 mg/kg/d at 7 d before CME and intraperitoneally injected with LY294002 10 mg/kg at 30 min before CME.

### Cardiac-function measurements

In our preliminary study, cardiac function was the worst at 6 h after CME [30]. We therefore measured the following parameters of cardiac function at 6 h after CME: left ventricular ejection fraction (LVEF), left ventricular end-diastolic diameter (LVEDd), fractional shortening (FS), and cardiac output (CO). A Hewlett Packard Sonos 7500 ultrasound instrument (Philips Technologies, USA) was used for the cardiac-function measurements. The probe frequency was 12 MHz. All measurements were average values of three cardiac cycles. Echocardiography was performed by an experienced specialist.

### Serum c-troponin I measurement

Blood samples (1.0 mL) were obtained from the femoral veins of the rats at 6 h after the sham operation or CME prior to sacrifice, and serum c-troponin I (cTnI) levels were calculated using the electrochemistry method according to the manufacturer’s instructions (Roche Inc., Switzerland).

### Tissue sampling and sample treatments

After the measurement of cardiac function, 2-3 mL of 10% potassium chloride was injected *via* the caudal vein to make the heart stop in diastole. The heart was harvested, and the auricular appendixes and atria were removed. The ventricles were divided into apical and basal parts by making an incision in the middle of the long axis of the left ventricle, parallel with the atrioventricular groove. The apical part was quickly frozen with liquid nitrogen and stored in a refrigerator at -80°C. This sample was subsequently used for fluorescent quantitative polymerase chain reaction and western blotting. The basal part was fixed with 4% paraformaldehyde for 12 h, embedded in paraffin, and sliced at a thickness of 4 μm. The paraffin-embedded samples were used for TUNEL assay to detect cardiomyocyte apoptosis and for hematoxylin-eosin (HE) staining and hematoxylin-basic fuchsin-picric acid (HBFP) staining to observe myocardial microinfarct areas.

### Measurement of myocardial microinfarct areas

HBFP staining is an important staining method to diagnose myocardial ischemia at the early stage. With this technique, ischemic cardiomyocytes are stained red, and the cytoplasm and nuclei of normal cardiomyocytes are stained yellow and blue, respectively. Each HBFP-stained slice was observed (×100 magnification) under a DMR+Q550 pathological image analyzer (Leica, Wetzlar, Germany). Five fields were randomly chosen for the measurement of infarct areas using the plane method of the Leica Qwin software. The infarct percent was calculated by dividing the infarct area by the total observed area [31].

### Detection of cardiomyocyte apoptosis with TUNEL assay

TUNEL assays were performed according to the manufacturer’s instructions (Roche, USA). Under a light microscope, the nuclei of apoptotic cells appeared yellow (TUNEL positive). The number of apoptotic and total cardiomyocytes in the microinfarct, peri-infarct, and non-infarct areas were calculated from 40 randomly chosen non-overlapping areas in each slice (×400 magnification). The apoptosis rate was calculated as follows: apoptosis rate = the number of apoptotic cardiomyocytes/the total number of cardiomyocytes × 100% [32].

### Cell culture and treatments

In brief, hearts from 1-3-day-old Sprague-Dawley rats (Medical Experimental Animal Center, Guangxi Medical University, China) were dissected and placed in cold phosphate-buffered saline. Cardiomyocytes were isolated using a combination of collagenase type II (Sigma, USA) and trypsin. The cardiomyocytes were pre-plated twice for 1 h each time to minimize contamination by non-myocytes and then cultured in Dulbecco modified Eagle medium (DMEM) with 20% fetal bovine serum (FBS; Gibco, Australia) at 37°C in a humidified incubator containing 5% CO_2_. The next day, the medium was replaced with DMEM supplemented with 10% FBS. The cardiomyocytes were ready to be treated as indicated after 2-3 days of culture. To achieve hypoxia, the plates were placed in a modular incubator chamber (Shanghai Stem Cell Technology Co.,Ltd., China) flushed with nitrogen at 20 L/min for 4 min to achieve an effective O_2_ level of < 3%. Gao et al. have shown that CME can be mimicked *in vitro* by culturing cardiomyocytes under hypoxic conditions for 12 h [33].

For the cell experiments, the cultured cardiomyocytes were divided into the normoxic control group (NC), 12-h hypoxia group (H12h), 12-h hypoxia + nicorandil group (Nic), 12-h hypoxia + nicorandil + LY294002 group (Nic+LY), and 12-h hypoxia + LY294002 group (LY). In the Nic group, 100 μM nicorandil was added to the cardiomyocytes 1 h before the hypoxia treatment. In the LY group, 10 μM LY294002 was added to the cardiomyocytes 1 h before the hypoxia treatment. In the Nic+LY group, 10 μM LY294002 and 100 μM nicorandil were added to the cardiomyocytes at 2 h and 1 h before the hypoxia treatment, respectively.

### Cell viability assay

Cardiomyocytes were plated at a density of 2.0 × 10^5^ cells/well in 96-well plates after the cell treatments. Cell viability was examined using Cell Counting Kit-8 (Dojindo Laboratory, Kumamoto, Japan) according to the manufacturer’s protocol. The number of surviving cells in the treatment groups was determined in duplicate relative to the number of untreated cells (NC group), which was taken as 100%.

### Western blot

Total proteins obtained from cardiac tissues and cardiomyocytes were separated using 10%-15% sodium dodecyl sulfate polyacrylamide gel electrophoresis and electrotransferred to polyvinylidene fluoride membranes (Millipore, Atlanta, GA, USA). The membranes were blocked with 5% bovine serum albumin or non-fat milk for 1.5 h at room temperature and then incubated overnight at 4°C with primary antibodies against p-PI3K, total PI3K, p-Akt, total Akt, Bax, Bcl-2, caspase-3, or GAPDH. All antibodies (1:1,000 dilution) were obtained from Cell Signaling Technology (Beverly, MA, USA). After being washed five times with Tris-buffered saline containing 0.1% Tween 20 (TBST), the membranes were incubated with secondary antibodies conjugated with horseradish peroxidase, in TBST for 2 h at room temperature. The signals were detected with an enhanced chemiluminescence detection system (Pierce, Rockford, IL, USA). The protein bands were assessed and quantified using Image Lab software from Bio-Rad.

### Statistical analysis

All values were expressed as mean ± SEM. Differences were compared using one-way analysis of variance. *P* < 0.05 was considered to be statistically significant. All statistical tests were performed with GraphPad Prism software version 5.0 (GraphPad Software Inc., San Diego, CA, USA).

## Abbreviations

CME: coronary microembolization
PCI: percutaneous coronary intervention
LVEF: left ventricular ejection fraction
FS: fractional shortening
CO: cardiac output
LVEDd: left ventricular end-diastolic diameter
PTEN: gene of phosphate and tension homology deleted on chromsome ten
cTnI: cardiac troponin I
HE: hematoxylin-eosin
HBFP: hematoxylin-basic fuchsin-picric acid
TUNEL: transferase-mediated deoxyuridine triphosphate-biotin nick end labeling
DMEM: Dulbecco modified Eagle medium
GAPDH: glyceraldehyde-3-phosphate dehydrogenase
TBST: tris-buffered saline with tween 20
